# Efficacy and Safety of Long-term Ketogenic Diet Therapy in a Patient With Type 1 Diabetes

**DOI:** 10.1210/jcemcr/luae102

**Published:** 2024-07-10

**Authors:** Andrew P Koutnik, Samuel Klein, Austin T Robinson, Joseph C Watso

**Affiliations:** Sansum Diabetes Research Institute, Santa Barbara, CA 93105, USA; Human Healthspan, Resilience, and Performance, Florida Institute for Human and Machine Cognition, Pensacola, FL 32502, USA; Sansum Diabetes Research Institute, Santa Barbara, CA 93105, USA; Center for Human Nutrition, Washington University School of Medicine, St. Louis, MO 63110, USA; Neurovascular Physiology Laboratory, School of Public Health, Indiana University Bloomington, Bloomington, IN 47405, USA; Cardiovascular & Applied Physiology Laboratory, Department of Health, Nutrition, and Food Sciences, Florida State University, Tallahassee, FL 32306, USA

**Keywords:** ketogenic diet, insulin, euglycemia, glycemic control

## Abstract

Fewer than 1% of patients with type 1 diabetes achieve normal glycemic control (glycated hemoglobin [HbA1c] < 5.7%/ < 39 mmol/mol). Additionally, exogenous insulin administration often causes “iatrogenic hyperinsulinemia,” leading to whole-body insulin resistance and increased risk of cardiovascular complications. We present data on the clinical efficacy and safety of a long-term (10-year) ketogenic diet (≤50 g carbohydrates/day) therapy in a patient with type 1 diabetes. The use of a ketogenic diet resulted in successful glycemic control, assessed by HbA1c (5.5%; 36.6 mmol/mol), continuous glucose monitoring median glucose (98 mg/dL; 5.4 mmol/L), and glucose time-in-range of 70 to 180 mg/dL (90%) without acute glycemic complications. In conjunction, there was a 43% decrease in daily insulin requirements. Low-density lipoprotein cholesterol increased, whereas small-dense low-density lipoprotein was in the normal range (<90 nmol/L). No adverse effects were observed on thyroid function, kidney function, or bone mineral density. This case report demonstrates that a long-term ketogenic diet in a person with type 1 diabetes has considerable therapeutic benefits.

## Introduction

Fewer than 1% of people with type 1 diabetes achieve normoglycemia (<5.7%/ < 39 mmol/mol glycated hemoglobin [HbA1c]) and only 20% achieve the recommended HbA1c target of <7.0% (<53 mmol/mol) (1). In addition, therapy with subcutaneous insulin injection bypasses the normal insulin delivery into the portal vein and causes “iatrogenic hyperinsulinemia,” which causes whole-body insulin resistance (2). Poor glycemic control and insulin resistance are important risk factors for cardiovascular disease. Patients with type 1 diabetes have a 10-fold higher risk of cardiovascular disease than the general population (3-6). Several case series, observational studies, and one 7-day randomized controlled trial have demonstrated that a ketogenic diet, defined as ≤50 g of carbohydrates per day (7) can reduce glycemic variability, HbA1c, and total daily insulin requirement in people with type 1 diabetes (8). However, the use of ketogenic diets in people with type 1 diabetes could also have adverse effects by causing hypoglycemia, diabetic ketoacidosis, increased plasma low-density lipoprotein (LDL) cholesterol, altered thyroid function, decreased renal function, and decreased bone mineral density (8-12). The purpose of this case report is to present data on the long-term (10-year) efficacy and safety of therapy with a ketogenic diet in a patient with type 1 diabetes.

## Case Presentation

The data provided in this case report were obtained from the patient's medical and personal records from 2013 to 2023, which was approved by the institutional review board for Human Subjects Research at Florida State University (institutional review board #4141). The patient was a male between the age of 30 to 35 years who developed type 1 diabetes on July 2006 when the patient was in middle adolescence (15-17 years old) and presented with diabetic ketoacidosis (blood glucose 590 mg/dL/32.7 mmol/L, pH 7.19, and HCO_3_ 7.9 mmol/L/7.9 mEq/L). Plasma glutamic acid decarboxylase autoantibody concentration was 222.6 U/mL (normal reference range: 0.0-5.0 U/mL) and C-peptide concentration was <0.1 ng/mL/<0.03 nmol/L (normal reference range: 1.1-4.4 ng/mL). The current report also includes more detailed information on body composition (measured via dual-energy x-ray absorptiometry [2013 GE Lunar DPX-IQ; 2023 GE Lunar iDXA; Madison, WI, USA]), daily insulin use, and clinical blood tests obtained in 2013 when the patient was between 20 and 25 years of age when he was on a diet based on the recommendations set by the American Diabetes Association (13) and again in 2023 when the patient was between 30 and 35 years of age after exactly 10 years of therapy with a ketogenic diet.

## Diagnostic Assessment

Daily dietary intake and physical activity were tracked for 60 days in 2013 and 2023 using a commercially available food tracker (Cronometer, Revelstoke, BC, Canada) and physical activity log (type, duration, intensity). A detailed analysis of glycemic control (assessed by using continuous glucose monitoring [Dexcom G6, San Diego, CA, USA]) and lipid profile (assessed by using nuclear magnetic resonance and immunologic analyses) were obtained in 2023. The patient did not habitually use any medications other than exogenous insulin from June 2006 to July 2023.

## Treatment

### Dietary Intake and Physical Activity

The patient consumed the American Diabetes Association-recommended diet individualized for dietary and physical activity needs (13), composed predominantly of whole food sources for 84 months from July 2006 to July 2013 after the diagnosis of type 1 diabetes was made and began to consume a ketogenic diet in July 2013 (Table 1). The ketogenic diet contained ≤50 g/day of carbohydrates including animal (eg, poultry, eggs, fish/seafood, cheese, full-fat dairy, whey) and plant-based (eg, tofu, tempeh, pea) protein, nonstarchy leafy vegetables high in fiber, nuts, seeds, and oils (eg, olive, coconut) (7). Compliance with the ketogenic diet (49.0 ± 18.8 g carbohydrates/day) was confirmed by a mean plasma β-hydroxybutyrate of 0.8 mmol/L (range: 0.3-1.5 mmol/L; Keto-Mojo, Napa, CA, USA) during 60-day monitoring in 2023. The patient maintained the same moderate-vigorous physical activity routine, consisting of resistance and aerobic exercise, before and after changing to the ketogenic diet.

**Table 1.** Diet, physical activity, body composition, and clinical characteristics before and after ketogenic diet therapy

	Regular diet (≤2013)	Ketogenic diet (2013-2023)	Normal range
**Dietary intake**			
Calories	3117 kcal/day	2936 kcal/day	3000 kcal/day
	(13 042 kJ/day)	(12 284 kJ/day)	(12 552 kJ/day)
Carbohydrate	35% kcal/day	7% kcal/day	45%-65% kcal/day
	(35% kJ/day)	(7% kJ/day)	(45%-65% kJ/day)
Fat	28% kcal/day	68% kcal/day	20%-35% kcal/day
	(28% kJ/day)	(68% kJ/day)	(20%-35% kJ/day)
Protein	37% kcal/day	25% kcal/day	10%-35% kcal/day
	(37% kJ/day)	(25% kJ/day)	(10%-35% kJ/day)
Fiber	40.0 g/day	34.0 g/day	30.8 g/day
	(0.0400 kg/day)	(0.0340 kg/day)	(0.0308 kg/day)
Saturated fat	24 g/day	65 g/day	n/a
	(0.0240 kg/day)	(0.0650 kg/day)	
Saturated fat	7% kcal/day	20% kcal/day	10% kcal/day
	(7% kJ/day)	(20% kJ/day)	(10% kJ/day)
**Physical activity**			
MVPA	540 minutes/week	577 minutes/week	>150 minutes/week
	(32 400 seconds/week)	(34 620 seconds/week)	(9000 seconds/week)
**Body composition**			
Body mass	102.3 kg	99.9 kg	n/a
	(102.3 kg)	(99.9 kg)	
Body mass index	26.8 kg/m^2^	26.1 kg/m^2^	<30.0 kg/m^2^
	(26.8 kg/m^2^)	(26.1 kg/m^2^)	(<30.0 kg/m^2^)
Waist-to-hip ratio	0.86	0.83	<0.95
	(86%)	(83%)	(<95%)
Body fat	9.1%	9.8%	8.0%-21.3%
	(9.1%)	(9.8%)	(8.0%-21.3%)
**Glycemic control and insulin therapy**			
HbA1c	6.8%	5.5%	4.0%-5.6%
	(51 mmol/mol)	(37 mmol/mol)	(20-38 mmol/mol)
Total daily dose	67 IU	38 IU	n/a
	(67 IU)	(38 IU)	
Relative insulin dose	0.65 IU/kg	0.38 IU/kg	n/a
	(0.65 IU/kg)	(0.38 IU/kg)	
Total basal dose	35 IU	32 IU	n/a
	(35 IU)	(32 IU)	
Total bolus dose	27 IU	6 IU	n/a
	(27 IU)	(6 IU)	
Basal:bolus ratio	56.5%/43.5%	84.5%/15.5%	n/a
	(56.5%/43.5%)	(84.5%/15.5%)	
**Lipids**			
Triglycerides	60 mg/dL	69 mg/dL	0-149 mg/dL
	(0.68 mmol/L)	(0.78 mmol/L)	(0-1.68 mmol/L)
HDL-C	88 mg/dL	86 mg/dL	>39 mg/dL
	(2.28 mmol/L)	(2.23 mmol/L)	(1.01 mmol/L)
LDL-C	69 mg/dL	129 mg/dL	0-99 mg/dL
	(1.79 mmol/L)	(3.34 mmol/L)	(0-2.56 mmol/L)
Total cholesterol	169 mg/dL	227 mg/dL	100-199 mg/dL
	(4.38 mmol/L)	(5.88 mmol/L)	(2.59-5.15 mmol/L)
**Thyroid function**			
TSH	0.936 mIU/L	0.736 mIU/L	0.450-4.500 mIU/L
	(0.936 uIU/mL)	(0.736 uIU/mL)	(0.450-4.500 uIU/mL)
T4, free (Direct)	1.24 ng/dL	1.41 ng/dL	0.82-1.77 ng/dL
	(0.016 nmol/L)	(0.018 nmol/L)	(0.011-0.023 nmol/L)
**Renal function**			
eGFR	103 mL/min/1.73 m^2^	102 mL/min/1.73 m^2^	>59 mL/min/1.73 m^2^
	(1.72 mL/s/1.73 m^2^)	(1.70 mL/s/1.73 m^2^)	(>0.99 mL/s/1.73 m^2^)
**Bone mineral density**			
Total body BMD	1.425 g/cm^2^	1.423 g/cm^2^	1.250 g/cm^2^)
	(0.001425 kg/cm^2^)	(0.001423 kg/cm^2^)	(0.00125 kg/cm^2^)

Values in parentheses are International System of Units (SI).

Abbreviations: BMD, bone mineral density; eGFR, estimated glomerular filtration rate; HbA1c, glycated hemoglobin; HDL-C, high-density lipoprotein cholesterol; LDL-C, low-density lipoprotein cholesterol; MVPA, moderate-to-vigorous physical activity; n/a, not applicable; T4, thyroxine.

### Body Composition

The patient's body mass was stable from 2013 to 2023 (Table 1), with <5% fluctuation throughout the 10-year period. Detailed body composition measurements confirmed consistent waist-to-hip ratio and body fat percentage in 2013 and 2023.

## Outcome and Follow-up

### Glycemic Control

HbA1c, assessed every 6 months, was 6.7% (49.7 mmol/mol) and 6.9% (51.9 mmol/mol) from January to June 2013 and decreased to 5.6% (37.7 mmol/mol) 3 months after starting the ketogenic diet. The participant's HbA1c values from 2013 to 2023 averaged 5.5% (Table 1; 36.6 mmol/mol). In addition, a 60-day continuous glucose monitor assessment in 2023 showed median glucose of 98 mg/dL (5.4 mmol/L), 90% time between 70 and 180 mg/dL (3.9-10.0 mmol/L), 83% between 70 and 140 mg/dL (3.9-7.8 mmol/L), 71% between 70 and 120 mg/dL (3.9-6.7 mmol/L), 0% time with glucose >250 mg/dL (13.9 mmol/L), 0% of time with glucose >180 mg/dL (10 mmol/L), 9% time with glucose <70 mg/dL (<3.9 mmol/L), and 0% time with glucose <54 mg/dL (Table 2; <3.0 mmol/L). There were no hypoglycemic or hyperglycemic events that required medical attention or any episodes of diabetic ketoacidosis from 2013 to 2023. Analyses of hypoglycemic awareness (14) in 2023 demonstrated intact hypoglycemic awareness.

**Table 2.** Continuous glucose monitoring results after ketogenic diet therapy

Variable	Ketogenic diet (2023)
Median glucose	98 [15] mg/dL
	(5.4 mmol/L [0.8 mmol/L])
Standard deviation glucose	23 [11] mg/dL
	(1.3 mmol/L [0.6 mmol/L])
Coefficient of variance glucose	24% [8]
	(24% [8])
Time-in-range 70-180 mg/dL	90% [11]
	(90% [11])
Time-in-tight-range 70-140 mg/dL	83 ± 10%
	(83 ± 10%)
Time-in-normoglycemia 70-120 mg/dL	71 ± 12%
	(71 ± 12%)
<54 mg/dL	0% [3]
<70 mg/dL	9% [11]
	(9% [11])
>180 mg/dL	0% [0]
	(0% [0])
>250 mg/dL	0% [0]
	(0% [0])

Values in parentheses are International System of Units (SI). Normally distributed data is presented as mean ± SD. Nonnormally distributed data is presented as median [interquartile range].

### Insulin use

Total insulin load decreased by 43% (from 0.67 IU/kg/day in 2013 to 0.38 IU/kg/day in 2023) during ketogenic diet therapy (Table 1). The decrease in insulin use was due to a decrease in bolus insulin and a marked shift in the percent of total insulin given as a basal dose (from 57% to 85%) and the percent given as a bolus dose (from 44% to 16%).

### Safety Assessment

It has been proposed that a ketogenic diet has adverse effects on plasma LDL cholesterol concentrations, thyroid and renal function, and bone mineral density (8-10, 12, 15). Although plasma triglyceride and high-density lipoprotein cholesterol concentrations did not change during 10 years of ketogenic diet therapy, plasma LDL cholesterol increased by 87% (from 69 to 129 mg/dL; 3.8 to 7.2 mmol/L), and total cholesterol increased by 34% (from 169 to 227 mg/dL; 1.9 mmol/L to 2.6 mmol/L) (Table 1). A detailed analysis of plasma lipids in 2023 revealed the increase in plasma LDL cholesterol was unlikely to have been caused by an increase in small dense LDL, which was within the normal range (<90 nmol/L), suggesting a plasma lipid pattern that is not associated with a significantly increased risk of cardiovascular disease (16, 17). TSH and free thyroxine 4 concentrations remained within the normal range during ketogenic diet therapy, and a more detailed thyroid profile in 2023 demonstrated free triiodothyronine (2.6 pg/mL), reverse T3 (serum: 18.6 ng/dL), and thyroxine binding globulin (19 µg/mL) were within the normal range. Estimated glomerular filtration rate, plasma creatinine concentration, and total body bone mineral density did not change after 10 years of ketogenic diet therapy, despite their increased age.

## Discussion

**Figure 1.**
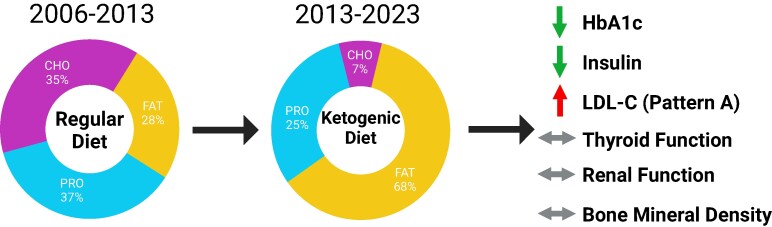
Safety and efficacy of long-term ketogenic diet therapy in a patient with type 1 diabetes. Long-term (10 years) therapy with a ketogenic diet (≤50 g carbohydrates/day) markedly improved glycemic control (HbA1c from 6.8% [50.8 mmol/mol] to 5.5% [36.6 mmol/mol]; 43% decrease in insulin requirements) without acute glycemic complications (severe hypoglycemic or diabetic ketoacidosis event) in a patient with type 1 diabetes. Although plasma total cholesterol increased 34% and LDL cholesterol increased 87%, the concentration of small dense LDL was within the normal range (<90 nmol/L). All other plasma lipids remained unchanged. No adverse effects on thyroid function, kidney function, or bone mineral density were detected.

This case report demonstrates that long-term (10-year) therapy with a ketogenic diet in a person with type 1 diabetes has considerable therapeutic benefits by causing a marked improvement in glycemic control (mean HbA1c decreased from 6.8% [50.8 mmol/mol] to 5.5% [36.6 mmol/mol]) and a 43% decrease in insulin requirements without adverse effects (Fig. 1). The sustained improvement in glycemia and decrease in insulin load-enhanced whole-body insulin sensitivity will likely decrease the risk of adverse microvascular and macrovascular outcomes (2, 3). This case demonstrates that long-term ketogenic diet therapy in people with type 1 diabetes is possible and has a dual metabolic benefit by improving glycemic control in conjunction with a decrease in insulin use, independent of any changes in body mass/composition, total daily energy intake, or physical activity.

The patient did not have any of the purported potential adverse effects of consuming a ketogenic diet (8-12). Although plasma LDL cholesterol concentration nearly doubled, an assessment of lipoprotein particle size showed this was likely caused by an increase in large buoyant LDL cholesterol (pattern A), which is not associated with an increased risk of atherosclerosis (16, 17). These findings are consistent with prior literature showing that carbohydrate restriction results in a dose-dependent reduction in small dense LDL independent of weight loss (18). The overall lipid profile (low plasma triglyceride, high high-density lipoprotein cholesterol, and normal small dense LDL), suggests ketogenic diet consumption did not significantly increase the risk of cardiovascular disease despite the increased plasma LDL cholesterol concentration. There were no changes in thyroid or kidney function, or bone mineral density despite the increase in age. Even though the patient had a greater percentage of time below range (9% time <70 mg/dL/<3.9 mmol/L) than is recommended by international consensus guidelines in type 1 diabetes (<4% time <70 mg/dL/<3.9 mmol/L) (19), the patient spent 0% at level 2 hypoglycemia (<54 mg/dL/<3.0 mmol/L). There were also no hypoglycemia or hyperglycemia events requiring medical attention. The patient presented with intact hypoglycemic awareness (14), suggesting that very low glycemic variability on a ketogenic diet did not result in any adverse events despite elevated time in hypoglycemia. Although there have been no prospective trials linking hypoglycemia and cardiovascular events (20), many have speculated on the acute cardiovascular risk of hypoglycemia following the progression of arterial and cardiac damage following multiple years of chronic hyperglycemia (21, 22). It is unclear how hypoglycemia will impact patients with chronic normoglycemia or nonpathologically elevated ketone bodies. Ketone bodies have been demonstrated to be cardioprotective (23, 24) and neuroprotective (25, 26) in normal physiologic levels, supplementing energy metabolism in low glucose environments, requiring further investigation.

This case report highlights the clinical benefits of a ketogenic diet as a primary therapy in a patient with type 1 diabetes. The positive experience of this patient underscores the need for randomized controlled trials to fully investigate the therapeutic effects, safety, and acceptability of a ketogenic diet in people with type 1 diabetes.

## Learning Points

- Fewer than 1% of patients with type 1 diabetes achieve normal glycemic control (HbA1c < 5.7%/<39 mmol/mol) and exogenous insulin administration often causes “iatrogenic hyperinsulinemia,” leading to whole-body insulin resistance and increased risk of cardiovascular complications.
- Long-term (10-year) ketogenic diet therapy in people with type 1 diabetes is possible and has a dual metabolic benefit by improving glycemic control 10-year mean HbA1c 5.5%/36.6 mmol/mol) in conjunction with a 43% decrease in insulin use, independent of changes in body mass/composition, total daily energy intake, or physical activity.
- Long-term (10-year) ketogenic diet therapy had no adverse effects on thyroid function, kidney function, or bone mineral density, with no hypoglycemic or hyperglycemic events that required medical attention or any episodes of diabetic ketoacidosis.
- Clinical efficacy and safety of a 10-year ketogenic diet in type 1 diabetes underscores the need for randomized controlled trials to thoroughly investigate the therapeutic effects, safety, and acceptability of a ketogenic diet in people with type 1 diabetes.

## Contributors

A.P.K. and J.C.W. collected and organized longitudinal data and developed the first draft. A.P.K., S.K., A.T.R., and J.C.W. provided critical input into the manuscript and data, approved the final version of the manuscript, and agree to be accountable for all aspects of the work in ensuring that questions related to the accuracy or integrity of any part of the work are appropriately investigated and resolved. A.P.K., S.K., A.T.R., and J.C.W. qualify for authorship, and all those who qualify for authorship are listed.

## Data Availability

Some or all datasets generated during and/or analyzed during the current study are not publicly available but are available from the corresponding author on reasonable request.
